# Pharmaceutical considerations in treating neuropathic pain in athletes

**DOI:** 10.1007/s00264-025-06440-4

**Published:** 2025-02-12

**Authors:** Sina Ramtin, Asif Ilyas

**Affiliations:** 1 Rothman Orthopaedics, Philadelphia, USA; 2 Drexel University, Philadelphia, USA

**Keywords:** Neuropathic pain, Athletes, Pain, Opioids, Steroids, Pain

## Abstract

Neuropathic pain is a complex and challenging condition that arises from abnormal processing of somatosensory information, often following nerve injury or dysfunction. Its diagnosis involves a detailed clinical history, sensory examination, and diagnostic tests such as electromyography, nerve conduction studies, and MRI to identify nerve damage or structural causes. In athletes, neuropathic pain can result from nerve entrapment syndromes, post-surgical complications, or peripheral nerve injuries, with unique challenges in pain assessment due to psychological factors and exercise-induced changes. Pharmacological management primarily includes anticonvulsants (e.g., gabapentin, pregabalin) and antidepressants (e.g., tricyclics, SNRIs), tailored to minimize side effects that could impair athletic performance. Effective treatment requires a careful balance to manage pain while maintaining physical capabilities. When treating athletes for neuropathic pain, healthcare providers must ensure prescribed medications comply with World Anti-Doping Agency (WADA) regulations. Narcotics (opioids) and cannabinoids are prohibited in-competition. Glucocorticoids are also banned in-competition if administered via injection, orally, or rectally, and elevated levels in urine may lead to sanctions.

## Introduction

### Neuropathic pain definition

Pain is often challenging to research due to its subjective nature, the complex interplay of factors influencing its generation and modulation, and the difficulty of objectively measuring its intensity [1]. Neuropathic pain arises from abnormal processing of somatosensory information generated within the body and its structures, rather than from external stimuli. It results from injury to the nervous system, which may be caused by trauma, toxic substances, or metabolic conditions affecting neurons either peripherally or centrally. Presentation can vary both between peripheral and central etiologies, as well as among individuals with the same etiology [2].

### Diagnosis of NP

The diagnosis of neuropathic pain begins with a detailed clinical evaluation, focusing on the patient’s history and symptoms. A thorough history helps identify key factors such as pain onset, characteristics (burning, stabbing, shooting), distribution, and any associated symptoms like sensory changes (numbness, tingling, allodynia, or hyperalgesia). The physical examination assesses neurological deficits, sensory abnormalities, and motor function. Red flags like progressive symptoms, systemic signs, or neurological deficits may signal more serious underlying conditions. The diagnostic criteria for neuropathic pain, such as those from the Neuropathic Pain Special Interest Group (NeuPSIG) [3] and the International Association for the Study of Pain (IASP) [4], combine clinical symptoms, positive sensory signs, and evidence of nerve injury.

In addition to the clinical assessment, diagnostic tests are often used to confirm the diagnosis or rule out other conditions. Electromyography and nerve conduction studies help identify peripheral neuropathy or nerve damage, while MRI can detect structural causes like spinal cord compression or nerve root injury. Blood tests rule out systemic causes such as diabetes or infections, while specific imaging or serology may be used to confirm conditions like postherpetic neuralgia or diabetic neuropathy. Proper differentiation from musculoskeletal, visceral, or vascular pain is crucial, as neuropathic pain presents unique qualities like burning or electric shock-like sensations.

## Neuropathic pain athletes

In athletes, pain assessment is further complicated by psychological and motivational factors, as well as exercise-induced changes in pain thresholds and tolerance. Chronic pain has long been misinterpreted, frequently seen as a result of tissue damage from acute trauma or overuse injuries [5]. Sports physicians must navigate the challenge of assessing the athlete’s reported pain by evaluating its chronicity, determining whether it is nociceptive, neuropathic, or a combination of both, and identifying the physiological processes responsible for the symptoms [6].

Neuropathic pain in athletes can arise from injuries, repetitive strain, or medical conditions affecting the nervous system. The most common ones can be classified into the following groups:

1. Nerve Entrapment Syndromes

Median nerve dysfunction manifesting as carpal tunnel syndrome can occur in athletes who perform repetitive wrist movements or sustain prolonged weight-bearing on their wrists. It is particularly prevalent in cyclists and motorcyclists due to prolonged pressure on the wrist while gripping handlebars, tennis players as a result of repeated wrist flexion and extension during strokes, weightlifters and gymnasts from load-bearing on the wrists during exercises such as bench presses, pull-ups, and pommel horse routines, and rowers due to the repetitive gripping of oars combined with wrist flexion.

Athletes who engage in repetitive elbow flexion, wrist pressure, or trauma to the elbow or wrist are at higher risk of ulnar nerve dysfunction manifesting as cubital tunnel syndrome. This includes throwing athletes like baseball pitchers and javelin throwers, cyclists from prolonged wrist pressure, golfers and tennis players from overuse during swings, weightlifters from strain during heavy lifts, and gymnasts from wrist hyperextension and elbow stress.

Runners, due to repetitive hip extension, flexion, and rotation, may experience piriformis tightness or spasm, increasing the risk of sciatic nerve dysfunction. Cyclists can develop tightness from prolonged sitting, especially with poor posture. Gymnasts and dancers, who perform deep stretches and frequent hip rotations, are also at risk. Football and rugby players may strain the piriformis muscle from sudden direction changes and repeated hip rotation. Weightlifters can experience tightness from lower-body movements that require strong hip activation, such as squats and deadlifts.

2. Post-Surgical Neuropathic Pain

Post-surgical neuropathic pain can include post-surgical neuralgia, where athletes may experience nerve-related pain at the surgical site after procedures like ACL reconstruction or shoulder surgery. Chronic Regional Pain Syndrome (CRPS) may also develop in rare cases following surgery or injury to a limb.

3. Nerve injuries

Peripheral nerve injuries can include Burner or Stinger Syndrome [7], which results from sudden nerve trauma in the neck or shoulder, commonly seen in contact sports like football or rugby. Axillary nerve injuries can occur after shoulder dislocations, often in football or wrestling. Peroneal nerve injuries, causing foot drop, are frequently caused by lateral knee trauma in sports like soccer or skiing.

Spinal nerve root compression can lead to lumbar radiculopathy (sciatica) due to herniated discs or spondylolisthesis, particularly in weightlifters or rowers. Cervical radiculopathy can occur in wrestlers or athletes with neck injuries.

Trauma-induced neuropathic pain may involve brachial plexus injuries from traumatic shoulder impacts, common in football or hockey, and nerve lacerations resulting from direct trauma in sports such as cycling accidents or fencing injuries.

## Pharmacotherapy for neuropathic pain

When managing neuropathic pain in athletes, the choice of treatment must consider not only the effectiveness of the medication but also its potential impact on performance, recovery, and side effects that could interfere with athletic abilities.

1. Anticonvulsants

Anticonvulsants help control the abnormal electrical firing of neurons, which often causes pain from nerve injuries or damage. For athletes, the goal is to manage pain while ensuring that the medication does not impair physical performance, focus, or coordination. These medications achieve this by modulating sodium channels to prevent excessive nerve firing, thus reducing the risk of pain flare-ups during training or competition. They also enhance GABA activity, an inhibitory neurotransmitter, to dampen overactive nerve signals that contribute to persistent pain. Additionally, some anticonvulsants inhibit calcium channels, which can reduce the release of pain-inducing neurotransmitters and improve nerve function without hindering motor skills.

Gabapentin (Neurontin) helps reduce neuropathic pain by blocking excessive nerve activity. It is often used for conditions like radiculopathy as well as peripheral nerve compression. Common side effects include dizziness, sedation, weight gain, and peripheral oedema, which can impact stamina, concentration, and mobility. Gabapentin typically starts at 300 mg once daily or in divided doses. The dose is gradually increased to 900–1800 mg per day, divided into two to three doses, based on efficacy and tolerance. The maximum dose is usually 3600 mg/day. Athletes should start with a lower dose and gradually increase it to minimize sedation or dizziness, which can impact performance. It is important for athletes to monitor their response to the medication to ensure it does not interfere with training or competition.

Pregabalin (Lyrica) works similarly to gabapentin by binding to the α2δ subunit of calcium channels but has a more potent effect and faster onset [8]. It is effective for conditions like fibromyalgia, which athletes may experience from overuse or long-term strain. Pregabalin is usually taken in divided doses twice a day, with adjustments based on individual response. Side effects include sedation, dizziness, weight gain, and peripheral edema, which can interfere with muscle recovery, endurance, and agility. While pregabalin’s fast onset can offer quick pain relief, athletes should monitor its impact on performance during training or competition. Pregabalin typically starts at 75 mg twice daily. The dose can be increased to 150–300 mg per day, divided into two doses. The maximum dose is usually 600 mg/day, also divided into two doses. Pregabalin works quickly, and higher doses may offer faster relief, but athletes should be cautious about sedation and dizziness, which can affect reaction time and coordination. It is important to start with a lower dose and adjust as needed to minimize these effects.

Carbamazepine (Tegretol) works by stabilizing sodium channels to prevent the over-firing of nerves, making it effective for pain caused by nerve compression or irritation [9]. It is commonly used to treat conditions like trigeminal neuralgia, which may occur in athletes after facial injuries, and other neuropathic pain due to direct nerve trauma [10]. The drug is typically started at a low dose and gradually increased, with regular monitoring to check for potential side effects and drug interactions. Common side effects include dizziness, drowsiness, nausea, and rash, while more serious issues like liver toxicity and blood disorders require routine testing. Carbamazepine typically starts at 200 mg twice daily. The dose is increased by 200 mg every two to three days until reaching 800–1200 mg per day, usually divided into two doses. The maximum dose is typically 1600 mg/day, though some cases may go up to 2000 mg/day with caution. Carbamazepine is effective for nerve compression-related pain but can cause sedation and cognitive dulling, which may affect mental sharpness and reaction times.

Carbamazepine, lamotrigine, and topiramate are used less frequently in the treatment of neuropathic pain compared to gabapentin and pregabalin [11, 12]. While they can be effective for specific types of neuropathic pain, such as trigeminal neuralgia or neuropathies related to compression (in the case of carbamazepine), they tend to have a more complex side effect profile. These medications are also less well tolerated by athletes due to potential impacts on mental clarity, cognitive function, and coordination, which can interfere with training and competition. Therefore, they are generally considered second or third-line options, often prescribed when first-line treatments (gabapentin or pregabalin) do not provide adequate relief (Table 1).

**Table 1:** Anticonvulsant dosage and considerations in Neuropathic Pain

Anticonvulsant	Starting Dose	Titration	Max Dose	Considerations
Gabapentin	300 mg once daily or divided doses	Increase to 900–1800 mg/day in 2–3 doses	3600 mg/day	Start low, titrate slowly to avoid sedation and dizziness, which can impact performance.
Pregabalin	75 mg twice daily	Increase to 150–300 mg/day, divided doses	600 mg/day	May work quickly but can cause sedation and dizziness; titrate carefully to minimize interference with training.
Carbamazepine	200 mg twice daily	Increase by 200 mg every 2–3 days to 800–1200 mg/day	1600 mg/day	Sedation and cognitive dulling may affect mental sharpness; monitor side effects closely.

2. Antidepressants

Tricyclic Antidepressants (TCAs) are one of the most widely used classes of medications for managing neuropathic pain, particularly when other first-line treatments (e.g., anticonvulsants) are not effective or well-tolerated. TCAs work primarily by inhibiting the reuptake of serotonin and norepinephrine in the central nervous system. This action increases the levels of these neurotransmitters in the synaptic cleft, enhancing their effects on pain modulation pathways. Specifically, the serotonin-norepinephrine balance plays a key role in controlling pain signals transmitted via the spinal cord and the brain.

Amitriptyline (Elavil) is commonly used when athletes experience nerve compression, radiculopathy, or post-surgical pain after orthopedic procedures like ACL reconstruction [13, 14]. The initial dose usually starts at 10–25 mg at bedtime, with gradual increases based on tolerance and response. The therapeutic dose typically ranges from 50 to 150 mg per day.

Nortriptyline (Aventyl) is used for chronic pain management in athletes, particularly for conditions like CRPS, tendinopathy, or persistent pain after fractures. The starting dose is typically 25 mg before bed, with the therapeutic dose usually ranging between 50 and 100 mg per day [15].

SNRIs (Selective Serotonin-Norepinephrine Reuptake Inhibitors), such as Duloxetine and Venlafaxine, are effective for treating neuropathic pain in athletes, particularly when there is co-occurring depression or anxiety often resulting from chronic injuries or overuse [16, 17]. For Duloxetine, the starting dose is 30–60 mg per day (once daily), with an effective dose typically ranging from 60 to 120 mg per day, which can be increased based on tolerance. For Venlafaxine, the starting dose is 37.5–75 mg per day (divided dose), with an effective dose between 75 and 225 mg per day (divided into two doses), adjusted for side effects [18]. SNRIs help athletes by providing pain relief for chronic musculoskeletal pain, sciatica, fibromyalgia, and nerve pain while also enhancing mood, reducing anxiety, and aiding in stress management during recovery [19, 20]. They work by increasing serotonin and norepinephrine, which helps with both pain modulation and mood regulation. Athletes should be cautious of dizziness or nausea, which may interfere with training, and monitor blood pressure, especially for those in strength-based sports where elevated blood pressure could be a concern (Table 2).

**Table 2:** Antidepressants Dosage and Side effects in Neuropathic Pain

Drug Class	Drug Name	Indications for Neuropathic Pain	Starting Dose	Effective Dose	Common Side Effects
Tricyclic Antidepressants (TCAs)	Amitriptyline	- Chronic musculoskeletal pain - Sciatica - Post-surgical neuropathy	10–25 mg/day (at night)	50–100 mg/day	- Drowsiness - Dry mouth - Constipation - Weight gain
	Nortriptyline	- Chronic pain - Neuropathic pain (e.g., diabetic neuropathy)	10–25 mg/day (at night)	50–100 mg/day	- Drowsiness - Dry mouth - Constipation
Selective Serotonin-Norepinephrine Reuptake Inhibitors (SNRIs)	Duloxetine	- Chronic musculoskeletal pain - Sciatica - Fibromyalgia - Post-surgical neuropathy	30–60 mg/day (once daily)	60–120 mg/day	- Nausea - Dizziness - Insomnia - Increased BP
	Venlafaxine	- Chronic musculoskeletal pain - Sciatica - Fibromyalgia	37.5–75 mg/day (divided doses)	75–225 mg/day (divided doses)	- Nausea - Dizziness - Increased BP - Sexual dysfunction

3. Nonsteroidal anti-inflammatory drugs (NSAIDs)

NSAIDs work by inhibiting COX-1 and COX-2 enzymes to reduce inflammation and pain, but they are not the first-line treatment for neuropathic pain, which involves nerve injury and central sensitization. Non-selective NSAIDs (e.g., Ibuprofen, Naproxen) may help with inflammation from conditions like radiculopathy or entrapment neuropathies in athletes, but they are less effective for the burning or shooting pain typical of nerve damage. COX-2 selective NSAIDs (e.g., Celecoxib, Etoricoxib) may be used for inflammation in cases like nerve root inflammation, offering fewer gastrointestinal side effects, but they are still limited to addressing the inflammatory component and not the neuropathic pain itself. Table 3 provides an overview of various NSAIDs, their dosages, and corresponding indications.

**Table 3:** Nonsteroidal anti-inflammatory drugs (NSAIDs) dosage and indications in Neuropathic Pain

NSAIDs	Dosage	Indication
Ibuprofen	400–600 mg every 4–6 h (max: 3200 mg/day)	Short-term inflammation relief (e.g., radiculopathy, musculoskeletal inflammation, nerve root irritation)
Naproxen	250–500 mg twice daily (max: 1000 mg/day)	Chronic inflammation in cases of entrapment neuropathies or post-surgical inflammation in athletes
Aspirin	325–1000 mg every 4–6 h (max: 4000 mg/day)	Acute inflammatory pain (e.g., post-surgical nerve inflammation, musculoskeletal injuries)
Celecoxib (COX-2 inhibitor)	100–200 mg once or twice daily (max: 400 mg/day)	Reduced GI side effects with nerve root inflammation or soft tissue injuries involving nerve compression (e.g., sciatica)
Diclofenac	50–75 mg twice daily (max: 150 mg/day)	Localized inflammation (e.g., tennis elbow, carpal tunnel syndrome), helpful in reducing inflammation around compressed nerves

4. Topical Agents

Topical agents are an important class of treatments for managing neuropathic pain in athletes, especially when the pain is localized or if there is a need to avoid systemic side effects associated with oral medications. They are often used for conditions like localized nerve entrapment, post-surgical pain, or peripheral neuropathy.

Lidocaine patches are local anaesthetics that block sodium channels, providing targeted pain relief by preventing pain signal propagation along nerves [21]. They are primarily used for neuropathic pain from conditions like carpal tunnel syndrome and localized musculoskeletal pain from nerve compression or injury [22]. Applied directly to the painful area for 12 h, the patches are simple, non-invasive, and can be cut to fit the affected area. Side effects are minimal, mainly skin irritation. They are ideal for patients sensitive to systemic medications, though their effectiveness is limited to superficial, localized pain and not suitable for deep or widespread nerve pain.

Capsaicin, derived from chili peppers, depletes substance P, a neuropeptide involved in pain transmission, leading to reduced pain signaling over time [23]. High-concentration patches (8%) are applied in clinical settings for conditions like postherpetic neuralgia, diabetic neuropathy, osteoarthritis with neuropathic components, CRPS, and localized neuropathic pain [24, 25]. The patch is applied for 60 min, with effects lasting for months. Side effects include burning sensations and skin irritation, but systemic effects are minimal. Capsaicin offers long-term pain relief and reduces reliance on oral medications, making it beneficial for athletes, though its initial discomfort may deter consistent use.

Diclofenac, a topical NSAID, inhibits COX enzymes to reduce prostaglandin production, providing localized pain relief and reducing inflammation [26]. It is effective for osteoarthritis-related pain, soft tissue injuries, tendinitis, bursitis, and acute sports injuries [27]. Available as a gel or patch, it is applied three to four times daily directly to the painful area. Side effects are minimal and include skin irritation or photosensitivity. Topical application minimizes systemic side effects compared to oral NSAIDs, though it may be less effective for deep tissue pain and should not be used on open wounds. Consistent use is necessary for optimal relief.

5. Corticosteroid injections

Corticosteroid injections are a targeted and effective treatment for managing neuropathic pain in athletes, especially when caused by nerve compression, inflammation, or irritation. These injections work by reducing swelling, suppressing immune-mediated nerve damage, and lowering the sensitivity of pain-sensing nerve.fibres Common conditions treated include carpal tunnel syndrome, where the injection is administered into the carpal tunnel to alleviate pressure on the median nerve, and tarsal tunnel syndrome, where it targets the tibial nerve near the medial ankle. In cases such as ulnar nerve entrapment, injections are applied around the cubital tunnel at the elbow, while for sciatica, they are directed to the epidural space or piriformis muscle. For Morton’s neuroma, injections are placed into the intermetatarsal space, and in post-surgical nerve pain, they are applied near the surgical site to address persistent inflammation [28]. Chronic regional pain syndrome may require regional or epidural injections for relief [29]. Medications commonly used include corticosteroids such as methylprednisolone, triamcinolone, or dexamethasone, providing effects that last around four to six weeks, often combined with local anaesthetics like lidocaine or bupivacaine for immediate, short-term relief. While corticosteroid injections offer localized, potent relief with minimal systemic side effects, they are most effective when combined with physical therapy and efforts to address the underlying cause of the pain.

6. Opioids

Opioids are powerful medications commonly used for managing severe pain, including neuropathic pain, especially when other treatments such as NSAIDs, anticonvulsants, or antidepressants are ineffective [30, 31]. Despite their effectiveness in pain relief, opioids come with a high potential for dependence, tolerance, and various other significant side effects. As a result, they are typically reserved as a last-resort option for short-term use in neuropathic pain and are carefully monitored when prescribed to minimize the risks associated with long-term use.

Opioids work by binding to opioid receptors in both the central nervous system and peripheral nervous system. These receptors include mu, kappa, and delta receptors. Mu receptors are primarily responsible for pain relief, but also contribute to euphoria, respiratory depression, and the potential for addiction. Kappa receptors also provide analgesia but are associated with dysphoria and hallucinations. Delta receptors play a lesser role in pain relief but help modulate pain. By binding to these receptors, opioids block pain signal transmission and alter the emotional response to pain, providing relief but also carrying a risk of dependence and other side effects. Table 4 represents the information on common opioids used for neuropathic pain.

**Table 4:** Opioid dosage in Neuropathic Pain

Opioid	Formulation	Typical Doses
Morphine	Oral, IV, ER (Extended Release)	- Oral: 15–30 mg every 4 h - IV: 2–10 mg every 3–4 h
Oxycodone	Oral (Immediate Release or ER)	- Immediate release: 5–15 mg every 4–6 h - Extended release: 10–20 mg every 12 h
Hydrocodone	Oral (with acetaminophen)	− 5–10 mg every 4–6 h (with acetaminophen, e.g., Vicodin)
Hydromorphone	Oral, IV, ER	- Oral: 2–4 mg every 4–6 h - IV: 0.2-1 mg every 3–4 h
Codeine	Oral (with acetaminophen)	− 15–60 mg every 4–6 h (with acetaminophen, e.g., Tylenol 3)

Short-term opioid use in athletes is indicated for severe acute pain (e.g., post-surgery, flare-ups of neuropathic conditions) and breakthrough pain that does not respond to other treatments. Opioids should be prescribed for the shortest duration possible, typically three to seven days, to manage acute pain. Combining opioids with non-opioid therapies, such as NSAIDs, anticonvulsants (e.g., gabapentin), or physical therapy, can help minimize reliance on opioids. Additionally, adjunct medications like laxatives for constipation and anti-nausea drugs can manage common side effects. It is crucial to maintain clear documentation of the need for opioids, prescribed dosages, and any refills. Regular follow-up appointments are essential to monitor the patient’s progress and assess the risk of misuse.

## Medication and doping- key considerations for athletes

When treating athletes for neuropathic pain, it is crucial to ensure that prescribed medications align with anti-doping regulations established by the World Anti-Doping Agency (WADA). Narcotics (opioids) and cannabinoids are prohibited during in-competition periods, according to WADA’s guidelines.

The WADA guidelines highlight the widespread use of glucocorticoids for their anti-inflammatory effects in treating musculoskeletal injuries such as bursitis and arthritis. However, since 2022, glucocorticoids are only prohibited in-competition when administered via injection, orally, or rectally. Even if administered outside of competition, elevated glucocorticoid levels in urine samples could exceed the permissible thresholds, potentially leading to sanctions. It is essential for both athletes and their healthcare providers to be fully aware of these regulations to ensure compliance and avoid accusations of performance-enhancing drug use during competition [32, 33].

It is important to regularly consult the most current WADA Prohibited List and the regulations specific to each athlete’s sport. If a prohibited substance is medically necessary, a Therapeutic Use Exemption (TUE) application should be considered to prevent any anti-doping violations.

Additionally, caution should be exercised with substances like cannabidiol (CBD). Despite being marketed as tetrahydrocannabinol (THC)-free, some CBD products may contain trace amounts of THC, which is prohibited in-competition. Physicians should ensure that athletes use third-party tested CBD products to avoid accidental doping violations [32, 34].

## Conclusion

The general approach to managing neuropathic pain in athletes starts with first-line treatments like Gabapentin, Pregabalin, NSAIDs, and topical agents, which are often effective. If pain persists or is severe, second-line treatments such as SNRIs or opioids may be considered. Treatment plans should be individualized based on the athlete’s specific condition, pain level, and response to treatment, with careful monitoring for side effects to avoid complications such as dependency or interference with healing.

## Data Availability

No datasets were generated or analysed during the current study.
